# Effectiveness of Interventions to Improve Glycemic Control in US Asian and Pacific Islander Populations With Type 2 Diabetes: Systematic Review and Meta-Analysis

**DOI:** 10.2196/75751

**Published:** 2026-04-30

**Authors:** Jason Xiao, Jielu Yu, Erin M Staab, Nikita C Thomas, Wen Wan, Amber N Deckard, Andrew J Karter, Monica E Peek, Elbert S Huang, Neda Laiteerapong

**Affiliations:** 1 Pritzker School of Medicine, University of Chicago, Chicago, IL, United States; 2 Section of General Internal Medicine, Department of Medicine, University of Chicago, 5841 S Maryland Ave, Chicago, IL, 60637, United States, 1 773-702-3608; 3 Division of Research, Kaiser Permanente, Pleasanton, CA, United States

**Keywords:** type 2 diabetes, systematic review, meta-analysis, Asian American, Pacific Islander, nonpharmacologic intervention, hemoglobin A_1c_, HbA_1c_, glycemic control

## Abstract

**Background:**

Asian American and Pacific Islander populations are disproportionately affected by diabetes.

**Objective:**

The purpose of this study was to assess the efficacy of nonpharmacologic interventions in reducing hemoglobin A_1c_ (HbA_1c_) levels among Asian American or Pacific Islander individuals with type 2 diabetes.

**Methods:**

A systematic review was conducted using the PubMed, Scopus, PsycInfo, and CINAHL databases, covering studies published from 1985 to 2019. Eligible studies were randomized controlled trials that evaluated nonpharmacologic interventions in outpatient settings for adults with type 2 diabetes in the United States, with at least 50% Asian American or Pacific Islander participants. Data extraction and risk of bias assessment were independently performed by 2 reviewers for each trial. The Cochrane Collaboration risk of bias tool and the Grading of Recommendations, Assessment, Development, and Evaluation approach were used to evaluate quality. Random-effects meta-analyses were conducted to estimate pooled effect sizes.

**Results:**

A total of 1835 articles were screened, with 9 randomized controlled trials meeting the inclusion criteria, comprising 1492 participants, with a median follow-up duration of 6 (IQR 3-12) months. Interventions included diabetes self-management education, bilingual counseling, glucose or weight monitoring, motivational interviewing, and financial support, often tailored to the cultural context of the participants. Pooled analysis of the 9 trials demonstrated a significant average HbA_1c_ reduction of −0.39% (95% CI −0.64% to −0.14%; *I*²=65%). Seven studies were judged to have a low risk of bias, 1 study had some concerns, and 1 study was assessed as high risk. The overall strength of evidence was high.

**Conclusions:**

Nonpharmacologic interventions substantively reduced HbA_1c_ levels in Asian American or Pacific Islander individuals with type 2 diabetes, particularly when culturally and linguistically tailored.

## Introduction

Asian American and Pacific Islander (AAPI) persons are disproportionately affected by diabetes, with a higher prevalence of type 2 diabetes compared to White persons, even when individuals have similar BMI or waist circumference [1]. The high burden of disease becomes even more apparent when examining disaggregated diabetes prevalence across AAPI ethnic groups [1]. For example, in the United States, Filipino men have one of the highest type 2 diabetes rates among all ethnic groups (15.8%), and Korean women are 5 times more likely to be affected by type 2 diabetes compared to non-Hispanic White women [2].

As recommended by the American Diabetes Association, lifestyle and educational interventions are key components of diabetes care and empower patients to make informed health decisions in coping with their disease [3]. Such interventions have been shown to be highly effective in promoting diabetes knowledge and self-care behaviors. Diabetes self-management education, for example, has been shown to reduce hemoglobin A_1c_ (HbA_1c_) in racial or ethnic minority populations [4]. However, only 3 reviews of diabetes interventions have focused on AAPI populations, and they have been limited in terms of geography, heterogeneity, and AAPI subpopulations included [5-7]. This systematic review and meta-analysis focused on AAPI persons with type 2 diabetes in the United States and aimed to broadly investigate the effect of nonpharmacological interventions on HbA_1c_ outcomes.

## Methods

This review was registered with the International PROSPERO (CRD42019122625) database and has been reported in accordance with the PRISMA (Preferred Reporting Items for Systematic Reviews and Meta-Analyses) guidelines (Table S1 in Multimedia Appendix 1).

### Search Strategy

A systematic search of the PubMed, Scopus, PsycInfo, and CINAHL databases was conducted from 1985 to 2019 without language restrictions, using search terms related to type 2 diabetes, study design, English as a second language, race or ethnicity, and disparities (Table S2 in Multimedia Appendix 1). After duplicate search results were removed, article titles and abstracts were reviewed, and studies that did not meet the criteria were excluded. A full-text review was conducted for the remaining articles. At each stage, articles were reviewed by at least 2 team members. Gray literature was not included in the search.

### Inclusion and Exclusion Criteria

We included randomized controlled trials of nonpharmacological interventions intended for adults with type 2 diabetes in the United States and its territories. We excluded inpatient, nursing home, and emergency department–based interventions; drugs, devices, surgeries, procedures, and diets; and interventions focused on diabetes prevention, screening, or diagnosis. At least 50% of trial participants had to be Asian, Asian American, Native Hawaiian, or Pacific Islander. Trials had to be at least 3 months in duration and report HbA_1c_ as an outcome (Table S3 in Multimedia Appendix 1).

### Data Extraction

Data were extracted by 2 independent reviewers using a standardized template, with discrepancies settled by a third reviewer or discussion with the research team. We extracted study characteristics, participant demographics, and HbA_1c_ outcomes. We categorized each intervention by its level (eg, individual, interpersonal, or community) and domain (eg, behavioral, sociocultural, or health care system) of influence based on the National Institute on Minority Health and Health Disparities Research Framework [8].

Among the randomized controlled trials (RCTs), HbA_1c_ results were reported in any of these 3 ways: (1) a difference in difference between intervention and control groups, (2) a difference between treatment and control groups at a specific follow-up time point, and (3) an HbA_1c_ value in a group at a follow-up time point (without group comparison). In the meta-analysis, we used 1 HbA_1c_ result per study, prioritizing the type of HbA_1c_ using this order.

### Quality Review

The Cochrane Collaboration risk of bias tool for RCTs was used to assess the risk of bias for each study [9]. Quality of evidence across trials was assessed using the Grading of Recommendations, Assessment, Development, and Evaluation approach. Quality of evidence (ie, degree of confidence that estimated effect approximates true effect) was rated high, moderate, low, or very low based on risk of bias, inconsistency, imprecision, indirectness, and publication bias. A funnel plot and Egger test were used to assess publication bias.

### Statistical Analysis

We conducted a random-effects meta-analyses and calculated a weighted average of the estimated effects within individual studies (a pooled effect estimate). We determined weights by the inverse of the within-study variance and between-studies variance for each study. We then constructed a *z* test and its associated 95% CI of pooled intervention effects. Heterogeneity was assessed with the *I*^2^ statistic. To assess consistency of findings, we used the same methods to perform subgroup analyses based on study populations (ie, Asian, Pacific Islander, and non-English language preferred).

## Results

**Figure 1.**
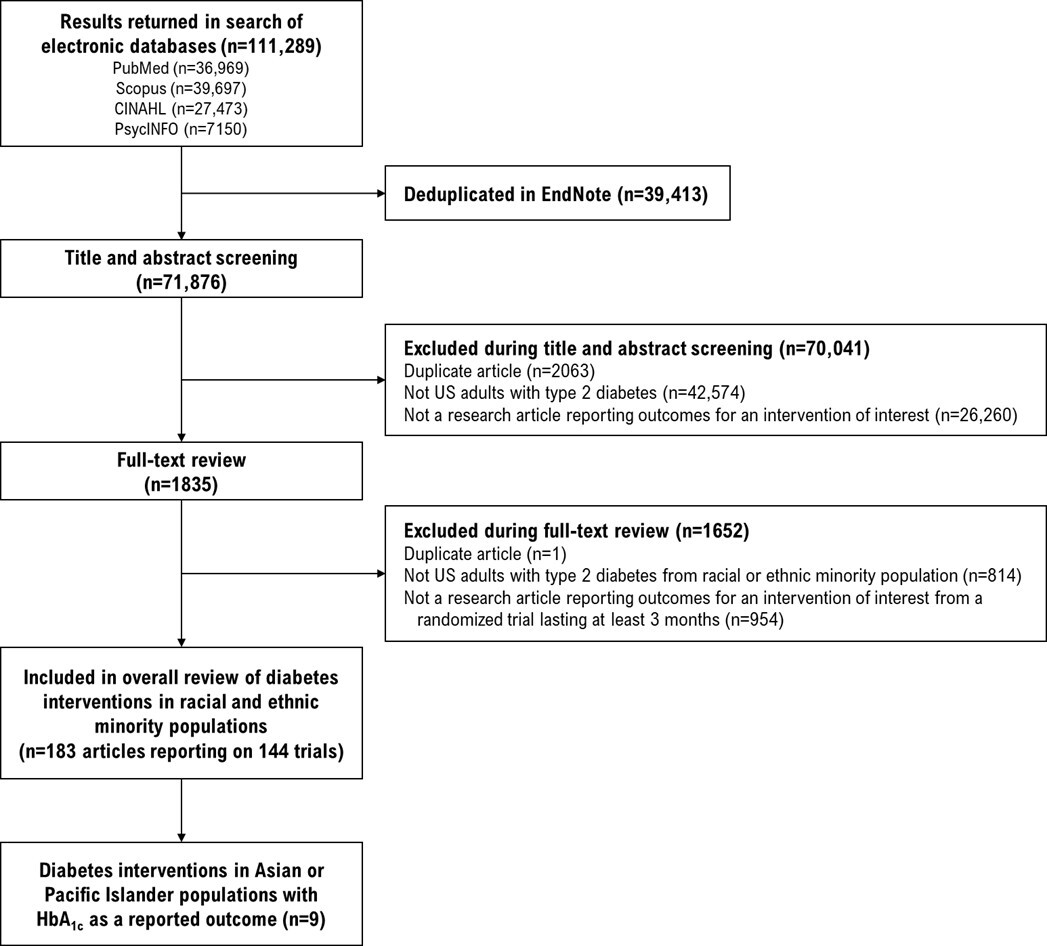
PRISMA (Preferred Reporting Items for Systematic Reviews and Meta-Analyses) flow diagram for the systematic review of randomized controlled trials of nonpharmacological interventions among Asian American and Pacific Islander populations with type 2 diabetes (1985‐2019). HbA_1c_: hemoglobin A_1c_.

Database searches returned 111,289 results. After removing duplicate results and screening titles and abstracts, 1835 articles remained for full-text review. Ultimately, 9 RCTs met all inclusion criteria (Figure 1) [10-18]. These enrolled a total of 1492 patients who were primarily aged between 40 and 60 years (Table 1); 5 RCTs included all AAPI patients. Specific races or ethnicities included Filipino, Samoan, Hawaiian, Micronesian, Bangladeshi, and Korean. Two RCTs only assessed whether patients identified as Asian or Pacific Islander.

**Table 1.** Randomized controlled trials of nonpharmacological interventions among Asian American and Pacific Islander populations with type 2 diabetes (1985‐2019).

Study	Location	Enrolled/ screened, n/N (%)	Arms and participant characteristics	Length of follow-up
Bender et al [10], 2017	San Francisco, California	45/113, 39.8%	Intervention: culturally adapted mHealth^a^ weight loss lifestyle intervention with feedback, coaching, and support (level: individual and interpersonal; domain: behavioral and sociocultural) Mean age 58 (SD 10) years 60% female 100% Filipino Control: waitlist Mean age 57 (SD 10) years 63% female 100% Filipino	3 mo
DePue et al [11], 2013	American Samoa	268/406, 66%	Intervention: culturally adapted education and care coordination delivered by nurse-community health worker team (level: individual and interpersonal; domain: behavioral, sociocultural, and health care system) Mean age 56 (SD 13) years 57% female 100% Samoan 66% preferred language Samoan Control: waitlist Mean age 54 (SD 13) years 65% female 100% Samoan 80% preferred language Samoan	12 mo
Fernandes et al [12], 2018	Hawaii	320/793, 40.3%	Intervention: financial incentives for diabetes management among Medicaid recipients (level: individual; domain: behavioral) Mean age 49 (SD 11) years 55% female 34% Native Hawaiian or Pacific Islander, 25% multiple races, 20% Asian, 17% White, and 6% Hispanic or Latino Control: usual care Mean age 48 (SD 10) years 53% female 33% multiple races, 30% Native Hawaiian or Pacific Islander, 22% Asian, 14% White, and 9% Hispanic or Latino	12 mo
Ing et al [13], 2016	Oahu, Hawaii	47/65, 72.3%	Intervention: bimonthly community facilitator– and health professional–led support group meetings as follow-up after a culturally tailored, community-based educational intervention (level: individual and interpersonal; domain: behavioral and sociocultural) Mean age 55 (SD 11) years 40% female 56% Native Hawaiian, 32% Micronesian, and 8% Filipino Control: bimonthly postcards reminders about diabetes self-management as follow-up Mean age 54 (SD 9) years 62% female 59% Native Hawaiian and 36% Micronesian	3 mo
Islam et al [14], 2018	New York, New York	336/880, 38.1%	Intervention: culturally adapted, community health worker–led educational intervention including 5 group sessions and 2 one-on-one sessions (level: individual; domain: behavioral and sociocultural) Mean age 54 (SD 11) years 40% female 100% Bangladeshi 55% limited English proficiency Control: one culturally adapted community health worker–led educational session Mean age 56 (SD 10) years 40% female 100% Bangladeshi 59% limited English proficiency	6 mo
Kim et al [15], 2009	Baltimore and Washington DC area	83/224, 37%	Intervention: culturally tailored education, home glucose monitoring with tele-transmission, and nurse telephone counseling (level: individual; domain: behavioral and sociocultural) Mean age 57 (SD 8) years 51% female 100% Korean Predominantly Korean speaking Control: waitlist Mean age 56 (SD 8) years 38% female 100% Korean Predominantly Korean speaking	6 mo
Kim et al [16], 2015	Baltimore and Washington DC area	250/597, 41.8%	Intervention: community-based, culturally tailored, multimodal behavioral intervention including group education sessions and coaching by nurses and community health workers (level: individual; domain: behavioral and sociocultural) Mean age 59 (SD 8) years 41% female 100% Korean 61% see a Korean-speaking doctor Control: waitlist Mean age 58 (SD 9) years 45% female 100% Korean 59% see a Korean-speaking doctor	12 mo
Ratanawongsa et al [17], 2014	San Francisco, California	362/910, 39.7%	Intervention: culturally tailored, automated telephone self-management support and health coaching intervention (level: individual and interpersonal; domain: behavioral, sociocultural, and health care system) Mean age 57 (SD 8) years 77% female 61% Asian or Pacific Islander, 25% Latino, 6% Black, and 6% White 54% Cantonese speaking and 20% Spanish speaking Control: waitlist Mean age 55 (SD 9) years 71% female 62% Asian or Pacific Islander, 20% Latino, 10% Black, and 7% White 55% Cantonese speaking and 18% Spanish speaking	6 mo
Sinclair et al [18], 2013	Hawaii	82/91, 90.1%	Intervention: culturally adapted, community-based educational intervention (level: individual and interpersonal; domain: behavioral and sociocultural) Mean age 53 (SD 12) years 63% female 100% Native Hawaiian, Pacific Islander, or Filipino Control: waitlist Mean age 55 (SD 10) years 62% female 100% Native Hawaiian, Pacific Islander, or Filipino	3 mo

a mHealth: mobile health.

Most RCTs (n=7) included a component of diabetes self-management education (DSME) in the intervention arm. Other strategies consisted of bilingual counseling (n=4), glucose or weight self-monitoring (n=3), motivational interviewing (n=1), and financial support (n=1). Approximately all (n=8) studies modified some element of their intervention toward the specific cultures of their study populations. Per the National Institute on Minority Health and Health Disparities Research Framework, studies were conducted at the individual (n=9) and interpersonal levels of influence (n=5), targeting primarily behavioral (n=9) and sociocultural domains (n=8). For controls, 6 RCTs used waitlist patients, 2 used a minimal intensity intervention, and 1 used usual care.

**Figure 2.**
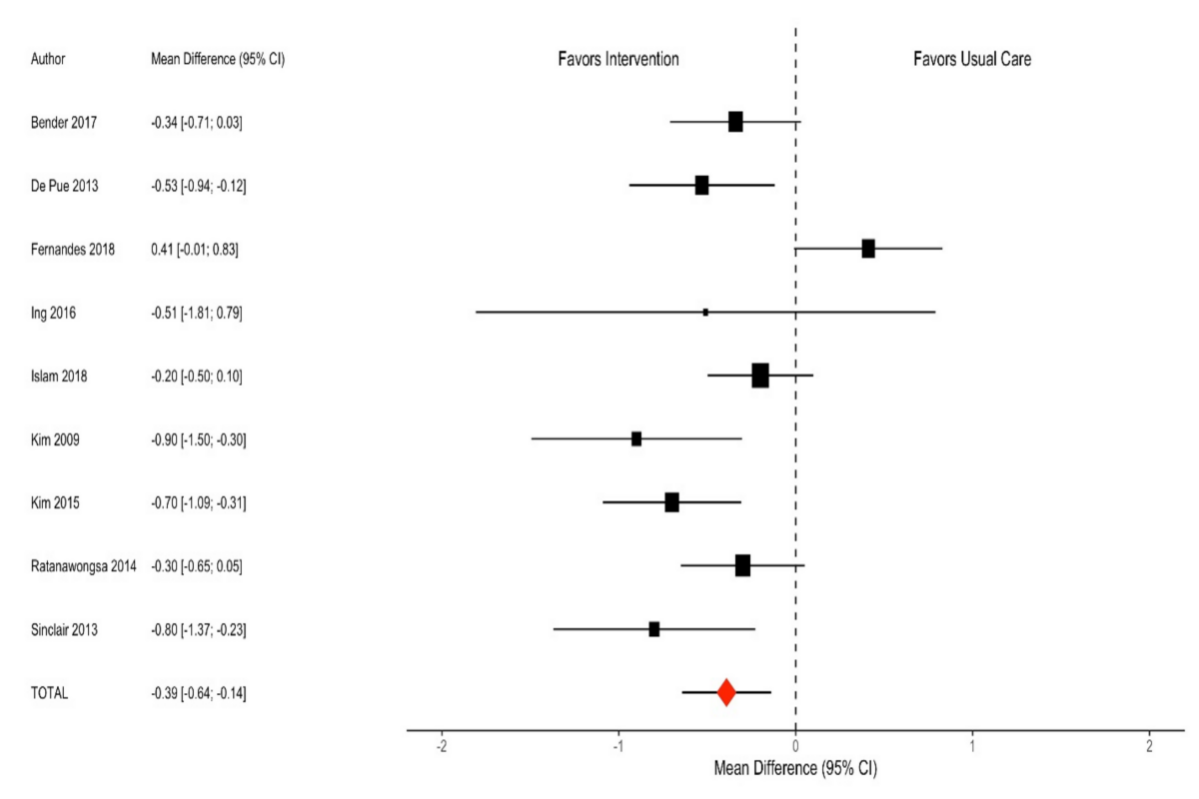
Forest plot of hemoglobin A_1c_ outcomes in nonpharmacological intervention trials among Asian American and Pacific Islander populations with type 2 diabetes (1985‐2019) [10-18].

Overall, individuals were followed for a median of 6 (range 3‐12) months. The interventions on average resulted in a −0.39% (95% CI −0.64 to −0.14%; *I*^2^=65%; Figure 2; Table 2) decrease in HbA_1c_. A similar pattern was observed with subgroup analyses for Asian individuals (n=5), with an average reduction in HbA_1c_ by −0.43% (95% CI −0.66 to −0.20%; *I*^2^=43%) and for Pacific Islander individuals (n=3), with a decrease of −0.62 % (95% CI −0.94 to −0.29%; *I*^2^=0%). For individuals who preferred non-English language (n=5), these interventions on average decreased HbA_1c_ levels by −0.47% (95% CI −0.70% to −0.23%; *I*^2^=45%). The most effective interventions specifically described using culturally tailored and/or language-tailored modalities of DSME.

**Table 2.** Meta-analysis of effects of nonpharmacological interventions on hemoglobin A_1c_ (HbA_1c_) outcomes among Asian American and Pacific Islander populations with type 2 diabetes (1985‐2019).

	Trials, n (%)	HbA_1c_^a^, weighted mean difference (95% Cl)	*I*^2^ statistic (%)
Overall			
Asian or Pacific Islander	9 (100)	−0.39 (−0.64 to −0.14)	65
Subgroup			
Asian	5 (56)	−0.43 (−0.66 to −0.20)	43
Pacific Islander	3 (34)	−0.62 (−0.94 to −0.29)	0
Non-English language preferred	5 (56)	−0.47 (−0.70 to −0.23)	45

a HbA_1c_: hemoglobin A_1c_.

Using the Cochrane Collaboration risk of bias for randomized trials tool, 7 of the 9 studies were determined to be at low risk of bias (Table S4 in Multimedia Appendix 1). One study [12] had some concerns in the domain of deviations from the intended intervention, and another study [17] was at high risk for bias in the domain of missing outcome data. Publication bias was evaluated visually by plotting changes in HbA_1c_ by study on a funnel plot, where the y-axis represents study size and the x-axis the size of effect found (Figure S1 in Multimedia Appendix 1). Of the 9 RCTs, 8 fell within the expected 95%, and approximate symmetry was achieved with the exception of the single study, whose intervention relied solely on financially incentivizing diabetes self-management. Additionally, 1 study with a small sample size (n=47) and relatively large HbA_1c_ SD at baseline affected the vertical distribution. The Egger test indicated no evidence of publication bias (*P*=.22). Quality of evidence was high by the GRADE (Grading of Recommendations, Assessment, Development, and Evaluation) approach (Table S5 in Multimedia Appendix 1).

## Discussion

We investigated the efficacy of nonpharmacologic interventions for type 2 diabetes in lowering HbA_1c_ in Asian American and Pacific Islander individuals. Our systematic review and meta-analysis identified a substantive reduction of HbA_1c_, with an average change of −0.39% over 6 months. Most interventions incorporated components of DSME and social engagement such as counseling or other interpersonal interactions. Furthermore, nearly all of the interventions tailored their approach based on the languages and cultures of their target population, often collaborating with community health workers or community-based organizations.

Identifying evidence-based ways to enhance diabetes management is essential for populations at greater risk for type 2 diabetes development and progression, including AAPI persons. AAPI populations have experienced alarming rises in diabetes; for instance, one study found a 68% increase in incidence between 1993 and 2001, the highest among all racial or ethnic groups [19]. Consequently, the American Diabetes Association has incorporated race or ethnicity into adult screening guidelines, and people with AAPI race or ethnicity are considered a “high-risk” population [20]. Furthermore, while AAPI race or ethnicity is comprised of many heterogeneous populations, current practices typically aggregate them as “Asian” or “Other,” when in reality over 40 ethnic subgroups fall under this categorization [21]. Encouragingly, in this review, few studies reported AAPI as a single group, and a broad range of AAPI subpopulations were represented (Filipino, Samoan, Hawaiian, Micronesian, Bangladeshi, and Korean). Heterogeneity was moderately high for overall results but lower in subgroup analyses. Data aggregation across AAPI subpopulations can mask higher-risk subpopulations and negatively impact screening, diagnosis, and treatment. For example, the DISTANCE study, which delineated AAPI subgroups when estimating diabetes rates, found that Pacific Islander, South Asian, and Filipino individuals had the highest prevalence of diabetes (18.3%, 15.9%, and 16.1%, respectively) across all racial or ethnic categories [22]. They also detected substantial differences in the rates of diabetes-related complications across AAPI subgroups [22].

AAPI persons constitute 7.7% of the US population and are one of the fastest growing racial or ethnic groups. Nonetheless, research funding for studies focusing on AAPI populations has not progressed over the past several decades. From 1986 to 2000, AAPI was represented in 0.2% of health-related grants across 7 federal agencies [23]. Between 1992 and 2018, 0.2% of the National Institutes of Health budget was allocated to 529 AAPI-related clinical research projects [24]. In the same time frame, inclusion of Native Hawaiians and Pacific Islanders in extramural National Institutes of Health–defined phase 3 clinical trials decreased from 0.3% (n=1011) to 0.2% (n=271) [24]. Given the small and shrinking number of RCTs among people with AAPI race or ethnicity, additional RCTs are needed to further our understanding of optimal strategies to manage type 2 diabetes in people with AAPI race or ethnicity.

Nonpharmacological interventions can meaningfully improve prevention and management of diabetes and have been found to be cost-effective; however, underrepresentation of AAPI persons in trials reduces generalizability [25-28]. For instance, the landmark Look Action for Health in Diabetes trial showed that intensive lifestyle interventions improved glycemic control and cardiovascular outcomes [29]. Although the Look Action for Health in Diabetes trial aimed to recruit at least a third of participants from racial and ethnic minority groups, there were only 50 (<2%) AAPI participants [30]. A meta-analysis of 16 randomized lifestyle intervention trials in general populations with type 2 diabetes found an average change of −0.37% in HbA_1c_ [31]. While this meta-analysis did not report stratified results or primarily enroll AAPI populations, their reduction in HbA_1c_ is very similar to our findings (−0.39%), providing some reassurance in the generalizability of larger studies.

Given the tremendous diversity of genetics, culture, diet, and lifestyle within the AAPI category, the effectiveness of specific interventions may vary by population. With our limited sample size, we were unable to directly compare types of interventions. However, we identified DSME as the most common shared component in these studies, which is one form of intervention that is strongly supported in the literature. In a systematic review of 118 randomized trials for adults with type 2 diabetes, DSME resulted in a −0.74% reduction in HbA_1c_ [32]. Furthermore, the study found that combining individual and group engagement resulted in greater reduction in HbA_1c_. However, studies that aggregate data at this level are unable to delineate racial or ethnic differences. Recently, different meta-analyses have demonstrated that nonpharmacologic interventions such as DSME may differ in effectiveness based on the population in focus. For example, in 2 meta-analyses, DSME interventions were shown to reduce HbA_1c_ (−0.24% and −0.25%) in Latino individuals with type 2 diabetes [33,34]. However, in another meta-analysis, DSME did not reduce HbA_1c_ in African American persons [35]. Mixed results have been reported in other meta-analyses, in which lifestyle interventions were effective in lowering HbA_1c_ for White individuals but not Black or African individuals or Hispanic populations [36]. Another systematic review found that lifestyle intervention reduced the incidence of type 2 diabetes more in the “(predominantly) White” group compared to the Asian group [37]. Moreover, beyond HbA_1c_, significant differences may exist in the impact on 2-hour glucose, BMI, and waist circumference depending on the racial or ethnic subgroup [38]. Taken together, these studies suggest that a uniform approach for nonpharmacologic management of type 2 diabetes would not lead to optimal health outcomes or reduction in health disparities.

In a report by the National Heart, Blood, and Lung Institute on improving cardiometabolic risk factors in Asian American individuals, the committee recommended more research to understand the social and cultural contexts of lifestyle interventions, perceptions, and barriers to addressing such risk factors [39]. This notion has taken root in the United States—for example, several studies focusing on South Asian individuals, such as the South Asian Healthy Lifestyle Intervention, use culturally tailored strategies to reduce cardiovascular risk factors [40]. Reviewing lifestyle interventions in South Asian individuals found that tailoring interventions to linguistic and cultural norms such as modifying traditional Indian cooking and incorporating physical activities from South Asia were effective in lowering HbA_1c_ [41].

In our study, 8 [10,11,13-18] of the 9 studies culturally tailored their intervention prior to implementation—for instance, Ing et al [13] in Hawaii created their intervention by partnering with local organizations and based their educational content on prior focus groups created in the community, highlighting relevant images, foods, and physical activities in Hawaii. In other studies, community health workers, who often are trusted members of the community, were employed to effectively carry out interventions in the study population. Overall, these studies highlight the potential benefits of actively partnering within and beyond traditional public health institutions, including community organizations and services, to bring culturally and linguistically appropriate care. Given the financial and logistical barriers to providing these more customized interventions to patients, telemedicine may be a promising approach. In the studies we reviewed, telephone counseling and remote blood glucose monitoring were strategies that proved effective in reducing HbA_1c_. Results of recent meta-analyses also support the effectiveness and potential cost-saving of telehealth and digital interventions for improving glycemic control [42-47]. Therefore, culturally adapting interventions, collaborating with community organizations, and using telemedicine and digital health tools are all possible strategies that health care systems can incorporate to improve healthy behaviors and outcomes for type 2 diabetes in AAPI communities.

This study has several limitations. First, a relatively small number of trials met all inclusion criteria for this meta-analysis. In part, this reflects a greater need for clinical trials to increase enrollment across underrepresented races or ethnicities, including those of AAPI backgrounds. While not feasible to conduct comparable studies by individual subgroup, stratifying AAPI populations by a limited number of diabetes and cardiovascular risk groups may prove useful to guide screening and management. Additionally, as with any meta-analysis, the dissimilarity of interventions makes differences in effect on HbA_1c_ difficult to compare. This is particularly true with nonpharmacologic interventions, given that each study developed a unique intervention for their populations. A larger sample size and further exploration of heterogeneity related to intervention approaches and study populations would be useful for understanding the generalizability of findings. Finally, it is possible that there are intervention studies published in the gray literature or indexed in other databases that were not included in this analysis.

In summary, this meta-analysis examined 9 nonpharmacologic interventions for type 2 diabetes for primarily AAPI populations. Random-effects meta-analysis found that such interventions substantively lowered HbA_1c_, offering support to leverage these interventions in clinical practice and community efforts. Successful interventions were culturally tailored and often used diabetes self-management education. More RCTs are needed to add to the small body of literature of effective nonpharmacologic interventions for AAPI populations with type 2 diabetes. Additionally, future studies are required to evaluate how other markers of cardiovascular risk and systemic health can be improved in these populations. Developing evidence-based approaches to managing type 2 diabetes in AAPI populations will further reduce the burden of diabetes in the United States and enhance quality of care.

## Supplementary material

10.2196/75751 Multimedia Appendix 1 Additional tables and figures: search terms, inclusion criteria, risk of bias, funnel plot, strength of evidence.

10.2196/75751 Checklist 1 PRISMA checklist.

## Abbreviations

AAPI: Asian American and Pacific Islander
DSME: diabetes self-management education
GRADE: Grading of Recommendations, Assessment, Development, and Evaluation
HbA_1c_: hemoglobin A_1c_
PRISMA: Preferred Reporting Items for Systematic Reviews and Meta-Analyses
RCT: randomized controlled trial
