# Supplementary material for: Effectiveness of Interventions to Improve Glycemic Control in US Asian and Pacific Islander Populations With Type 2 Diabetes: Systematic Review and Meta-Analysis
Source: Asian Pac Isl Nurs J. 2026 Apr 30;10:e75751. doi: 10.2196/75751 (PMC13132533; doi:10.2196/75751)
Supplement: Multimedia Appendix 1 [file apinj-v10-e75751-s001.docx]

Supplemental Table 1. PRISMA Checklist

| **Section and Topic** | **Item #** | **Checklist item** | **Location where item is reported (page)** |
| --- | --- | --- | --- |
| **TITLE** | | |  |
| Title | 1 | Identify the report as a systematic review. | 1 |
| **ABSTRACT** | | |  |
| Abstract | 2 | See the PRISMA 2020 for Abstracts checklist below. | 2 |
| **INTRODUCTION** | | |  |
| Rationale | 3 | Describe the rationale for the review in the context of existing knowledge. | 4 |
| Objectives | 4 | Provide an explicit statement of the objective(s) or question(s) the review addresses. | 4 |
| **METHODS** | | |  |
| Eligibility criteria | 5 | Specify the inclusion and exclusion criteria for the review and how studies were grouped for the syntheses. | 5, Supplemental Table 3 |
| Information sources | 6 | Specify all databases, registers, websites, organisations, reference lists and other sources searched or consulted to identify studies. Specify the date when each source was last searched or consulted. | 5 |
| Search strategy | 7 | Present the full search strategies for all databases, registers and websites, including any filters and limits used. | Supplemental Table 2 |
| Selection process | 8 | Specify the methods used to decide whether a study met the inclusion criteria of the review, including how many reviewers screened each record and each report retrieved, whether they worked independently, and if applicable, details of automation tools used in the process. | 5 |
| Data collection process | 9 | Specify the methods used to collect data from reports, including how many reviewers collected data from each report, whether they worked independently, any processes for obtaining or confirming data from study investigators, and if applicable, details of automation tools used in the process. | 5-6 |
| Data items | 10a | List and define all outcomes for which data were sought. Specify whether all results that were compatible with each outcome domain in each study were sought (e.g. for all measures, time points, analyses), and if not, the methods used to decide which results to collect. | 5-6 |
|  | 10b | List and define all other variables for which data were sought (e.g. participant and intervention characteristics, funding sources). Describe any assumptions made about any missing or unclear information. | 5-6 |
| Study risk of bias assessment | 11 | Specify the methods used to assess risk of bias in the included studies, including details of the tool(s) used, how many reviewers assessed each study and whether they worked independently, and if applicable, details of automation tools used in the process. | 6 |
| Effect measures | 12 | Specify for each outcome the effect measure(s) (e.g. risk ratio, mean difference) used in the synthesis or presentation of results. | 6 |
| Synthesis methods | 13a | Describe the processes used to decide which studies were eligible for each synthesis (e.g. tabulating the study intervention characteristics and comparing against the planned groups for each synthesis (item #5)). | 6-7 |
|  | 13b | Describe any methods required to prepare the data for presentation or synthesis, such as handling of missing summary statistics, or data conversions. | 6-7 |
|  | 13c | Describe any methods used to tabulate or visually display results of individual studies and syntheses. | 6-7 |
|  | 13d | Describe any methods used to synthesize results and provide a rationale for the choice(s). If meta-analysis was performed, describe the model(s), method(s) to identify the presence and extent of statistical heterogeneity, and software package(s) used. | 6-7 |
|  | 13e | Describe any methods used to explore possible causes of heterogeneity among study results (e.g. subgroup analysis, meta-regression). | 6-7 |
|  | 13f | Describe any sensitivity analyses conducted to assess robustness of the synthesized results. | 6-7 |
| Reporting bias assessment | 14 | Describe any methods used to assess risk of bias due to missing results in a synthesis (arising from reporting biases). | 6 |
| Certainty assessment | 15 | Describe any methods used to assess certainty (or confidence) in the body of evidence for an outcome. | 6 |
| **RESULTS** | | |  |
| Study selection | 16a | Describe the results of the search and selection process, from the number of records identified in the search to the number of studies included in the review, ideally using a flow diagram. | 7, Figure 1 |
|  | 16b | Cite studies that might appear to meet the inclusion criteria, but which were excluded, and explain why they were excluded. | n/a |
| Study characteristics | 17 | Cite each included study and present its characteristics. | 7, Table 1 |
| Risk of bias in studies | 18 | Present assessments of risk of bias for each included study. | Supplemental Table 4 |
| Results of individual studies | 19 | For all outcomes, present, for each study: (a) summary statistics for each group (where appropriate) and (b) an effect estimate and its precision (e.g. confidence/credible interval), ideally using structured tables or plots. | Figure 2 |
| Results of syntheses | 20a | For each synthesis, briefly summarise the characteristics and risk of bias among contributing studies. | 13 |
|  | 20b | Present results of all statistical syntheses conducted. If meta-analysis was done, present for each the summary estimate and its precision (e.g. confidence/credible interval) and measures of statistical heterogeneity. If comparing groups, describe the direction of the effect. | 12, Table 2, Figure 2 |
|  | 20c | Present results of all investigations of possible causes of heterogeneity among study results. | 12 |
|  | 20d | Present results of all sensitivity analyses conducted to assess the robustness of the synthesized results. | 12 |
| Reporting biases | 21 | Present assessments of risk of bias due to missing results (arising from reporting biases) for each synthesis assessed. | 13, Supplemental Figure |
| Certainty of evidence | 22 | Present assessments of certainty (or confidence) in the body of evidence for each outcome assessed. | 13, Supplemental Table 5 |
| **DISCUSSION** | | |  |
| Discussion | 23a | Provide a general interpretation of the results in the context of other evidence. | 14-18 |
|  | 23b | Discuss any limitations of the evidence included in the review. | 18 |
|  | 23c | Discuss any limitations of the review processes used. | 18 |
|  | 23d | Discuss implications of the results for practice, policy, and future research. | 14-18 |
| **OTHER INFORMATION** | | |  |
| Registration and protocol | 24a | Provide registration information for the review, including register name and registration number, or state that the review was not registered. | 4 |
|  | 24b | Indicate where the review protocol can be accessed, or state that a protocol was not prepared. | 4 |
|  | 24c | Describe and explain any amendments to information provided at registration or in the protocol. | n/a |
| Support | 25 | Describe sources of financial or non-financial support for the review, and the role of the funders or sponsors in the review. | 19 |
| Competing interests | 26 | Declare any competing interests of review authors. | 19 |
| Availability of data, code and other materials | 27 | Report which of the following are publicly available and where they can be found: template data collection forms; data extracted from included studies; data used for all analyses; analytic code; any other materials used in the review. | 19 |

**PRISMA Checklist for Abstracts**

| **Section and Topic** | **Item #** | **Checklist item** | **Reported (Yes/No)** |
| --- | --- | --- | --- |
| **TITLE** | | |  |
| Title | 1 | Identify the report as a systematic review. | Yes |
| **BACKGROUND** | | |  |
| Objectives | 2 | Provide an explicit statement of the main objective(s) or question(s) the review addresses. | Yes |
| **METHODS** | | |  |
| Eligibility criteria | 3 | Specify the inclusion and exclusion criteria for the review. | Yes |
| Information sources | 4 | Specify the information sources (e.g. databases, registers) used to identify studies and the date when each was last searched. | Yes |
| Risk of bias | 5 | Specify the methods used to assess risk of bias in the included studies. | Yes |
| Synthesis of results | 6 | Specify the methods used to present and synthesise results. | Yes |
| **RESULTS** | | |  |
| Included studies | 7 | Give the total number of included studies and participants and summarise relevant characteristics of studies. | Yes |
| Synthesis of results | 8 | Present results for main outcomes, preferably indicating the number of included studies and participants for each. If meta-analysis was done, report the summary estimate and confidence/credible interval. If comparing groups, indicate the direction of the effect (i.e. which group is favoured). | Yes |
| **DISCUSSION** | | |  |
| Limitations of evidence | 9 | Provide a brief summary of the limitations of the evidence included in the review (e.g. study risk of bias, inconsistency and imprecision). | Yes |
| Interpretation | 10 | Provide a general interpretation of the results and important implications. | Yes |
| **OTHER** | | |  |
| Funding | 11 | Specify the primary source of funding for the review. | No |
| Registration | 12 | Provide the register name and registration number. | No |

*From:*  Page MJ, McKenzie JE, Bossuyt PM, Boutron I, Hoffmann TC, Mulrow CD, et al. The PRISMA 2020 statement: an updated guideline for reporting systematic reviews. BMJ 2021;372:n71. doi: 10.1136/bmj.n71

Supplemental Table 2. Search Terms

| Database | Concept | Search terms |
| --- | --- | --- |
| CINAHL  *3/1/2019: 27,473 results* | *Diabetes* | ( ( ( ( ( (MH "Diabetes Mellitus+") OR (MH "Diabetes Education") OR (MH "Diabetes Educators") OR diabetes OR diabetic OR diabetics OR "noninsulin-dependent" OR "noninsulin dependent" OR "non-insulin-dependent" OR "non-insulin dependent" ) NOT ( (MH "Diabetes Insipidus") OR "diabetes insipidus" ) ) |
|  | *Study Design* | AND (LA English) AND (PY 1996-2019) NOT ( (TI "meta-analysis" OR TI "cross-sectional") ) ) NOT (PT systematic review OR PT doctoral dissertation OR PT masters thesis OR PT proceedings OR PT anecdote) |
|  | *Language* | AND (language OR languages OR English OR literacy OR "foreign-born" OR "foreign born" OR immigrant OR immigrants OR refugee OR refugees OR migrant OR migrants OR interpreter OR interpreters OR monolingual OR bilingual OR multilingual OR linguistic OR linguistically OR (MH "English as a Second Language") OR (MH "Language") OR (MH "Literacy") OR (MH "Illiteracy") |
|  | *Race/Ethnicity* | OR (MH "Immigrants+") OR (MH "Refugees") OR (MH "Race Relations+") OR (MH "Race Factors") OR (MH "Minority Groups") OR (MH "Ethnic Groups") OR (MH "Arabs") OR (MH "Asians+") OR (MH "Blacks") OR (MH "Hispanics") OR (MH "Eskimos+") OR (MH "Native Americans") OR (MH "Health Services, Indigenous") OR (MH "Transcultural Care") OR (MH "Cultural Competence") OR (MH "Cultural Sensitivity") OR cultural OR culturally OR multicultural OR multi-cultural OR transcultural OR trans-cultural OR crosscultural OR cross-cultural OR ethnic OR ethnicity OR ethnicities OR ethnically OR multiethnic OR multi-ethnic OR race OR races OR racial OR racially OR multiracial OR multi-racial OR biracial OR minority OR minorities OR "non-white" OR "non-whites" OR black OR blacks OR african OR hispanic OR hispanics OR latino OR latinos OR latina OR latinas OR latinx OR "puerto rican" OR "puerto ricans" OR "puerto rico" OR "mexican american" OR "mexican americans" OR "cuban american" OR "cuban americans" OR "native american" OR "native americans" OR "american indian" OR "american indians" OR "Indian Health Service" OR "alaska native" OR "alaska natives" OR "alaskan native" OR "alaskan natives" OR "native hawaiian" OR "native hawaiians" OR "pacific islander" OR "pacific islanders" OR "American Samoan" OR "American Samoans" OR "American Samoa" OR Guam OR Guamanian OR Guamanians OR Chamorro OR Chamorros OR "arab american" OR "arab americans" OR asian OR asians OR "Chinese American" OR "Chinese Americans" OR "Filipino American" OR "Filipino Americans" OR "Indian American" OR "Indian Americans" OR "people of color" |
|  | *Disparities* | OR (MH "Healthcare Disparities") OR (MH "Health Status Disparities") OR (MH "Health Services Accessibility") OR (MH "Social Determinants of Health") OR (MH "Community Health Centers") OR (MH "Safety-Net Providers") OR (MH "Community Health Services") OR (MH "Medically Underserved") OR (MH "Medically Underserved Area") OR (MH "Poverty+") OR (MH "Health Services for the Indigent") OR (MH "Urban Health Services") OR (MH "Medically Uninsured") OR equity OR inequity OR inequities OR equality OR inequality OR inequalities OR disparity OR disparities OR poor OR poverty OR vulnerable OR indigent OR underserved OR disadvantaged OR uninsured OR "publicly insured" OR medicaid OR "safety-net" OR "safety net" OR "health center" OR FQHC OR "community clinic" OR "free clinic" OR "low income" OR socioeconomic OR "social determinants") ) ) |
| PsycInfo  *3/1/2019: 7,150 results* | *Diabetes* | ( ( (SU "Diabetes Mellitus") OR (SU "Type 2 Diabetes") OR diabetes OR diabetic OR diabetics OR "noninsulin-dependent" OR "noninsulin dependent" OR "non-insulin-dependent" OR "non-insulin dependent" ) AND PY 1996-2019 AND LA English NOT ( (SU "Diabetes Insipidus") OR "diabetes insipidus") |
|  | *Study Design* | NOT ( TI "meta-analysis" OR TI "cross-sectional" OR MR "meta analysis" OR MR "metasynthesis" OR MR "systematic review" OR MR "scientific simulation" OR PT "dissertation abstract" OR (PT "encyclopedia") OR (PZ "abstract collection") OR (PZ "bibliography") OR (PZ "dissertation") OR (PZ "encyclopedia entry") OR (PZ "interview") OR (PZ "obituary") OR (PZ "poetry") OR (PZ "review-book") OR (PZ "review-media") OR (PZ "review-software & other") OR (PZ "column/opinion") or (PZ "editorial") OR (PZ "comment/reply") or (PZ "erratum/correction") OR (PZ "publication information") ) NOT ( ( PO Animal NOT ( PO Animal AND PO Human ) ) ) ) |
|  | *Language* | AND ( language OR languages OR (SU "English as Second Language") OR (SU "Language Proficiency") OR "English proficiency" OR "English speaking" OR "non-English" OR (SU "Literacy") OR literacy OR monolingual OR bilingual OR multilingual OR linguistic OR linguistically OR (SU "Interpreters") OR interpreter OR interpreters OR foreign-born OR "foreign born" |
|  | *Race/Ethnicity* | OR immigrant OR immigrants OR refugee OR refugees OR (SU "Migrant Farm Workers") OR (SU "Blacks") OR (SU "African Cultural Groups") OR black OR blacks OR african OR (SU "Alaska Natives") OR (SU "American Indians") OR "native american" OR "native americans" OR "american indian" OR "american indians" OR "Indian Health Service" OR "alaska native" OR "alaska natives" OR "alaskan native" OR "alaskan natives" OR (SU "Arabs") OR "arab american" OR "arab americans" OR (SU "Asians") OR asian OR asians OR "Chinese American" OR "Chinese Americans" OR "Filipino American" OR "Filipino Americans" OR "Indian American" OR "Indian Americans" OR (SU "Chinese Cultural Groups") OR (SU "Southeast Asian Cultural Groups") OR (SU "South Asian Cultural Groups") OR (SU "Latinos/Latinas") OR (SU "Mexican Americans") OR hispanic OR hispanics OR latino OR latinos OR latina OR latinas OR latinx OR "puerto rican" OR "puerto ricans" OR "puerto rico" OR "mexican american" OR "mexican americans" OR "cuban american" OR "cuban americans" OR (SU "Pacific Islanders") OR (SU "Hawaii Natives") OR "native hawaiian" OR "native hawaiians" OR "pacific islander" OR "pacific islanders" OR "American Samoan" OR "American Samoans" OR "American Samoa" OR Guam OR Guamanian OR Guamanians OR Chamorro OR Chamorros OR (SU "Racial and Ethnic Groups") OR ethnic OR ethnicity OR ethnicities OR ethnically OR multiethnic OR multi-ethnic OR race OR races OR racial OR racially OR multiracial OR multi-racial OR biracial OR minority OR minorities OR "non-white" OR "non-whites" OR "people of color" |
|  | *Disparities* | OR (SU "Cultural Sensitivity") OR (SU "Cross Cultural Treatment") OR (SU "Cross Cultural Communication") OR cultural OR culturally OR multicultural OR multi-cultural OR (SU "Health Disparities") OR equity OR inequity OR inequities OR equality OR inequality OR inequalities OR disparity OR disparities OR "health center" OR FQHC OR (SU "Community Health") OR "community clinic" OR "free clinic" OR "safety-net" OR "safety net" OR uninsured OR "publicly insured" OR Medicaid OR poor OR poverty OR indigent OR "low income" OR socioeconomic OR "urban health" OR vulnerable OR underserved OR disadvantaged OR "social determinants") |
| PubMed  *3/1/2019: 36,969 results* | *Diabetes* | "Diabetes Mellitus"[Mesh] OR diabetes[tiab] OR diabetic[tiab] OR diabetics[tiab] OR "noninsulin-dependent"[tiab] OR "noninsulin dependent"[tiab] OR "non-insulin-dependent"[tiab] OR "non-insulin dependent"[tiab]) NOT ("Diabetes Insipidus"[Mesh] or "diabetes insipidus" |
|  | *Study Design* | NOT ((("Animals"[Mesh] NOT ("Animals"[Mesh] AND "Humans"[Mesh])))))) AND "1996/01/01"[PDat] : "3000/12/31"[PDat])) NOT (meta-analysis[ti] or "cross-sectional"[ti])) NOT ((Address[pt] or Autobiography[pt] or Bibliography[pt] or Biography[pt] or "Case Reports"[pt] or "Clinical Conference"[pt] or "Clinical Trial, Veterinary"[pt] or "Clinical Trial Protocol"[pt] or Congress[pt] or "Consensus Development Conference"[pt] or dataset[pt] or "Consensus Development Conference, NIH"[pt] or "Dictionary"[pt] or "Dictionary"[pt] or "Duplicate Publication"[pt] or Editorial[pt] or "Expression of Concern"[pt] or Festschrift[pt] or "Government Document"[pt] or Guideline[pt] or "Interactive Tutorial"[pt] or Interview[pt] or Lecture[pt] or "Legal Case"[pt] or Legislation[pt] or "Meta-Analysis"[pt] or news[pt] or "Newspaper Article"[pt] or "Observational Study, Veterinary"[pt] or "Patient Education Handout"[pt] or Personal Narrative[pt] or Portrait[pt] or Practice Guideline[pt] or "Publication Components"[pt] or review[pt] or "Scientific Integrity Review"[pt] or "Study Characteristics"[pt] or "Systematic Review"[pt] or "Video-Audio Media"[pt] or Webcasts[pt])))) AND English[lang] |
|  | *Race/Ethnicity* | OR "Refugees"[Mesh] OR ("Ethnic Groups"[Mesh:NoExp] OR "African Americans"[Mesh] OR "American Native Continental Ancestry Group"[Mesh] OR "Asian Americans"[Mesh] OR "Oceanic Ancestry Group"[Mesh] OR "Arabs"[Mesh] OR "Hispanic Americans"[Mesh] OR "Race Factors"[Mesh] OR "Cultural Competency"[Mesh] OR "Culturally Competent Care"[Mesh] OR cultural[tw] OR culturally[tw] OR multicultural[tw] OR multi-cultural[tw] OR transcultural[tw] OR trans-cultural[tw] OR crosscultural[tw] OR cross-cultural[tw] OR ethnic[tw] OR ethnicity[tw] OR ethnicities[tw] OR ethnically [tw] OR multiethnic[tw] OR multi-ethnic[tw] OR race[tw] OR races[tw] OR racial[tw] OR racially[tw] OR multiracial[tw] OR multi-racial[tw] OR biracial[tw] OR minority[tw] OR minorities[tw] OR "non-white"[tw] OR "non-whites"[tw] OR black[tw] OR blacks[tw] OR african[tw] OR hispanic[tw] OR hispanics[tw] OR latino[tw] OR latinos[tw] OR latina[tw] OR latinas[tw] OR latinx[tw] OR "puerto rican"[tw] OR "puerto ricans"[tw] OR "puerto rico"[tw] OR "mexican american"[tw] OR "mexican americans"[tw] OR "cuban american"[tw] OR "cuban americans"[tw] OR "native american"[tw] OR "native americans"[tw] OR "american indian"[tw] OR "american indians" OR "indian health service"[tw] OR "United States Indian Health Service"[Mesh] OR "alaska native"[tw] OR "alaska natives"[tw] OR "alaskan native"[tw] OR "alaskan natives"[tw] OR "native hawaiian"[tw] OR "native hawaiians"[tw] OR "pacific islander"[tw] OR "pacific islanders"[tw] OR "american samoan"[tw] OR "american samoa"[tw] OR "american samoans"[tw] OR guam[tw] OR guamanian[tw] OR guamanians[tw] OR chamorro[tw] OR chamorros[tw] OR "arab american"[tw] OR "arab americans"[tw] OR asian[tw] OR asians[tw] OR "chinese american"[tw] OR "filipino american"[tw] OR "indian american"[tw] OR "people of color"[tw])) |
|  | *Language* | AND ((((Language[Mesh] OR language[tw] OR languages[tw] OR English[tw] OR Literacy[Mesh] OR literacy[tw] OR "foreign-born"[tw] OR "foreign born"[tw] OR immigrant[tw] OR immigrants[tw] OR refugee[tw] OR refugees[tw] OR interpreter[tw] OR interpreters[tw] OR monolingual[tw] OR bilingual[tw] OR multilingual[tw] OR linguistic[tw] OR linguistically[tw])) |
|  | *Disparities* | OR (Healthcare Disparities[Mesh] OR "Health Status Disparities"[Mesh] OR "Health Equity"[Mesh] OR "Social Determinants of Health"[Mesh] OR "Medically Underserved Area"[Mesh] OR "Medically Uninsured"[Mesh] OR "Health Services Accessibility"[Mesh:NoExp] OR "Urban Health"[Mesh] OR "Community Health Services"[Mesh:NoExp] OR "Community Health Centers"[Mesh:NoExp] OR "Safety-net Providers"[Mesh] OR "Poverty"[Mesh] OR equity[tw] OR inequity[tw] OR inequities[tw] OR equality[tw] OR inequality[tw] OR inequalities[tw] OR disparity[tw] OR disparities[tw] OR poor[tw] OR poverty[tw] OR vulnerable[tw] OR indigent[tw] OR underserved[tw] OR disadvantaged[tw] OR uninsured[tw] OR "publicly insured"[tw] OR medicaid[tw] OR "safety-net"[tw] OR "safety net"[tw] OR "health center"[tw] OR FQHC[tw] OR "community clinic"[tw] OR "free clinic"[tw] OR "low income"[tw])))) |
| Scopus  *3/1/2019: 39,697 results* | *Diabetes* | ( ( TITLE-ABS-KEY ( "diabetes" ) OR TITLE-ABS-KEY ( "diabetic" ) OR TITLE-ABS-KEY ( "noninsulin-dependent" ) OR TITLE-ABS-KEY ( "T2D" ) OR TITLE-ABS-KEY ( "non-insulin-dependent" ) ) AND NOT ( TITLE-ABS-KEY ( "diabetes insipidus" ) ) |
|  | *Study Design* | AND NOT ( TITLE ( "meta-analysis" ) OR TITLE ( "cross-sectional" ) ) AND NOT ( DOCTYPE ( ab ) OR DOCTYPE ( bk ) OR DOCTYPE ( ch ) OR DOCTYPE ( cr ) OR DOCTYPE ( er ) ) AND NOT ( ( KEY ( animal OR nonhuman ) ) AND NOT ( KEY ( animal OR nonhuman ) AND KEY ( human ) ) ) ) AND ( LIMIT-TO ( PUBYEAR , 2019 ) OR LIMIT-TO ( PUBYEAR , 2018 ) OR LIMIT-TO ( PUBYEAR , 2017 ) OR LIMIT-TO ( PUBYEAR , 2016 ) OR LIMIT-TO ( PUBYEAR , 2015 ) OR LIMIT-TO ( PUBYEAR , 2014 ) OR LIMIT-TO ( PUBYEAR , 2013 ) OR LIMIT-TO ( PUBYEAR , 2012 ) OR LIMIT-TO ( PUBYEAR , 2011 ) OR LIMIT-TO ( PUBYEAR , 2010 ) OR LIMIT-TO ( PUBYEAR , 2009 ) OR LIMIT-TO ( PUBYEAR , 2008 ) OR LIMIT-TO ( PUBYEAR , 2007 ) OR LIMIT-TO ( PUBYEAR , 2006 ) OR LIMIT-TO ( PUBYEAR , 2005 ) OR LIMIT-TO ( PUBYEAR , 2004 ) OR LIMIT-TO ( PUBYEAR , 2003 ) OR LIMIT-TO ( PUBYEAR , 2002 ) OR LIMIT-TO ( PUBYEAR , 2001 ) OR LIMIT-TO ( PUBYEAR , 2000 ) OR LIMIT-TO ( PUBYEAR , 1999 ) OR LIMIT-TO ( PUBYEAR , 1998 ) OR LIMIT-TO ( PUBYEAR , 1997 ) OR LIMIT-TO ( PUBYEAR , 1996 ) ) |
|  | *Language* | AND ( TITLE-ABS-KEY ( "language" ) OR TITLE-ABS-KEY ( "English" ) OR TITLE-ABS-KEY ( "literacy" ) OR TITLE-ABS-KEY ( "monolingual" ) OR TITLE-ABS-KEY ( "bilingual" ) OR TITLE-ABS-KEY ( "multilingual" ) OR TITLE-ABS-KEY ( "linguistic" ) OR TITLE-ABS-KEY ( "linguistically" ) OR TITLE-ABS-KEY ( "interpreter" ) AND ( LIMIT-TO ( LANGUAGE , "English" ) OR EXCLUDE ( LANGUAGE , "Spanish" ) OR EXCLUDE ( LANGUAGE , "Portuguese" ) OR EXCLUDE ( LANGUAGE , "French" ) OR EXCLUDE ( LANGUAGE , "Turkish" ) OR EXCLUDE ( LANGUAGE , "German" ) OR EXCLUDE ( LANGUAGE , "Italian" ) OR EXCLUDE ( LANGUAGE , "Polish" ) OR EXCLUDE ( LANGUAGE , "Arabic" ) OR EXCLUDE ( LANGUAGE , "Chinese" ) OR EXCLUDE ( LANGUAGE , "Croatian" ) OR EXCLUDE ( LANGUAGE , "Thai" ) OR EXCLUDE ( LANGUAGE , "Dutch" ) OR EXCLUDE ( LANGUAGE , "Korean" ) OR EXCLUDE ( LANGUAGE , "Japanese" ) OR EXCLUDE ( LANGUAGE , "Russian" ) OR EXCLUDE ( LANGUAGE , "Serbian" ) OR EXCLUDE ( LANGUAGE , "Czech" ) OR EXCLUDE ( LANGUAGE , "Persian" ) OR EXCLUDE ( LANGUAGE , "Slovak" ) OR EXCLUDE ( LANGUAGE , "Greek" ) OR EXCLUDE ( LANGUAGE , "Romanian" ) OR EXCLUDE ( LANGUAGE , "Slovenian" ) ) AND ( LIMIT-TO ( AFFILCOUNTRY , "United States" ) OR LIMIT-TO ( AFFILCOUNTRY , "Puerto Rico" ) OR LIMIT-TO ( AFFILCOUNTRY , "American Samoa" ) OR LIMIT-TO ( AFFILCOUNTRY , "Guam" ) OR LIMIT-TO ( AFFILCOUNTRY , "Virgin Islands (U.S.)" ) OR LIMIT-TO ( AFFILCOUNTRY , "Undefined" ) ) |
|  | *Race/Ethnicity* | OR TITLE-ABS-KEY ( "foreign-born" ) OR TITLE-ABS-KEY ( "immigrant" ) OR TITLE-ABS-KEY ( "refugee" ) OR TITLE-ABS-KEY ( "migrant" ) OR TITLE-ABS-KEY ( "black" ) OR TITLE-ABS-KEY ( "african" ) OR TITLE-ABS-KEY ( "native american" ) OR TITLE-ABS-KEY ( "american indian" ) OR TITLE-ABS-KEY ( "alaska native" ) OR TITLE-ABS-KEY ( "alaskan native" ) OR TITLE-ABS-KEY ( "indian health service" ) OR TITLE-ABS-KEY ( "arab american" ) OR TITLE-ABS-KEY ( "asian" ) OR TITLE-ABS-KEY ( "chinese american" ) OR TITLE-ABS-KEY ( "filipino american" ) OR TITLE-ABS-KEY ( "indian american" ) OR TITLE-ABS-KEY ( "hispanic" ) OR TITLE-ABS-KEY ( "latino" ) OR TITLE-ABS-KEY ( "latina" ) OR TITLE-ABS-KEY ( "latinx" ) OR TITLE-ABS-KEY ( "puerto rican" ) OR TITLE-ABS-KEY ( "puerto rico" ) OR TITLE-ABS-KEY ( "mexican american" ) OR TITLE-ABS-KEY ( "cuban american" ) OR TITLE-ABS-KEY ( "native hawaiian" ) OR TITLE-ABS-KEY ( "pacific islander" ) OR TITLE-ABS-KEY ( "american samoa" ) OR TITLE-ABS-KEY ( "american samoan" ) OR TITLE-ABS-KEY ( "guam" ) OR TITLE-ABS-KEY ( "guamanian" ) OR TITLE-ABS-KEY ( "chamorro" ) OR TITLE-ABS-KEY ( "ethnic" ) OR TITLE-ABS-KEY ( "ethnicity" ) OR TITLE-ABS-KEY ( "ethnicities" ) OR TITLE-ABS-KEY ( "ethnically " ) OR TITLE-ABS-KEY ( "multiethnic" ) OR TITLE-ABS-KEY ( "race" ) OR TITLE-ABS-KEY ( "racial" ) OR TITLE-ABS-KEY ( "racially" ) OR TITLE-ABS-KEY ( "multiracial" ) OR TITLE-ABS-KEY ( "biracial" ) OR TITLE-ABS-KEY ( "minority" ) OR TITLE-ABS-KEY ( "minorities" ) OR TITLE-ABS-KEY ( "non-white" ) OR TITLE-ABS-KEY ( "people of color" ) |
|  | *Disparities* | OR TITLE-ABS-KEY ( "cultural" ) OR TITLE-ABS-KEY ( "culturally" ) OR TITLE-ABS-KEY ( "multicultural" ) OR TITLE-ABS-KEY ( "transcultural" ) OR TITLE-ABS-KEY ( "crosscultural" ) OR TITLE-ABS-KEY ( "equity" ) OR TITLE-ABS-KEY ( "inequity" ) OR TITLE-ABS-KEY ( "inequities" ) OR TITLE-ABS-KEY ( "equality" ) OR TITLE-ABS-KEY ( "inequality" ) OR TITLE-ABS-KEY ( "inequalities" ) OR TITLE-ABS-KEY ( "disparity" ) OR TITLE-ABS-KEY ( "disparities" ) OR TITLE-ABS-KEY ( "safety-net" ) OR TITLE-ABS-KEY ( "health center" ) OR TITLE-ABS-KEY ( "FQHC" ) OR TITLE-ABS-KEY ( "community clinic" ) OR TITLE-ABS-KEY ( "free clinic" ) OR TITLE-ABS-KEY ( "uninsured" ) OR TITLE-ABS-KEY ( "publicly insured" ) OR TITLE-ABS-KEY ( "medicaid" ) OR TITLE-ABS-KEY ( "poor" ) OR TITLE-ABS-KEY ( "poverty" ) OR TITLE-ABS-KEY ( "indigent" ) OR TITLE-ABS-KEY ( "low income" ) OR TITLE-ABS-KEY ( "urban health" ) OR TITLE-ABS-KEY ( "vulnerable" ) OR TITLE-ABS-KEY ( "underserved" ) OR TITLE-ABS-KEY ( "disadvantaged" ) OR TITLE-ABS-KEY ( "social determinants" ) ) |

Supplemental Table 3. Inclusion Criteria According to PICOS Framework

|  | Criteria |
| --- | --- |
| Population | Adults ≥ 18 years  Type 2 diabetes  USA including territories  ≥ 50% Asian, Asian American, Native Hawaiian, or Pacific Islander (or stratified results reported for Asian, Asian American, Native Hawaiian, or Pacific Islander participants) |
| Intervention | Any non-pharmacological intervention* |
| Comparison | Any control comparison group (e.g., usual care, attention control, waitlist) |
| Outcome | Hemoglobin A1C |
| Study Design | Randomized controlled trial  ≥ 3 months duration |

*Based on NIH definition: An intervention is defined as a manipulation of the subject or subject’s environment for the purpose of modifying one or more health-related biomedical or behavioral processes and/or endpoints. Examples include: delivery systems (e.g., telemedicine, face-to-face interviews); strategies to change health-related behavior (e.g., cognitive therapy, exercise, development of new habits); and treatment strategies. From <https://grants.nih.gov/faqs#/clinical-trial-definition.htm?anchor=54901>. We excluded inpatient, nursing home, and emergency department based interventions; drugs, devices, surgeries, procedures, and diets; and interventions focused on diabetes prevention, screening, or diagnosis.

Supplemental Table 4. Risk of Bias

| **Study** | **Randomization Process** | **Deviations from Intended Intervention** | **Missing Outcome Data** | **Measurement of the Outcome** | **Selection of the Reported Results** | **Overall** |
| --- | --- | --- | --- | --- | --- | --- |
| Bender, 2017 | Low | Low | Low | Low | Low | Low |
| De Pue, 2013 | Low | Low | Low | Low | Low | Low |
| Fernandes, 2018 | Low | Some concerns | Low | Low | Low | Some concerns |
| Ing, 2016 | Low | Low | Low | Low | Low | Low |
| Islam, 2018 | Low | Low | Low | Low | Low | Low |
| Kim, 2009 | Low | Low | Low | Low | Low | Low |
| Kim, 2015 | Low | Low | Low | Low | Low | Low |
| Ratanawongsa, 2014 | Low | Low | High | Low | Low | High |
| Sinclair, 2013 | Low | Low | Low | Low | Low | Low |

Supplemental Figure. Funnel Plot


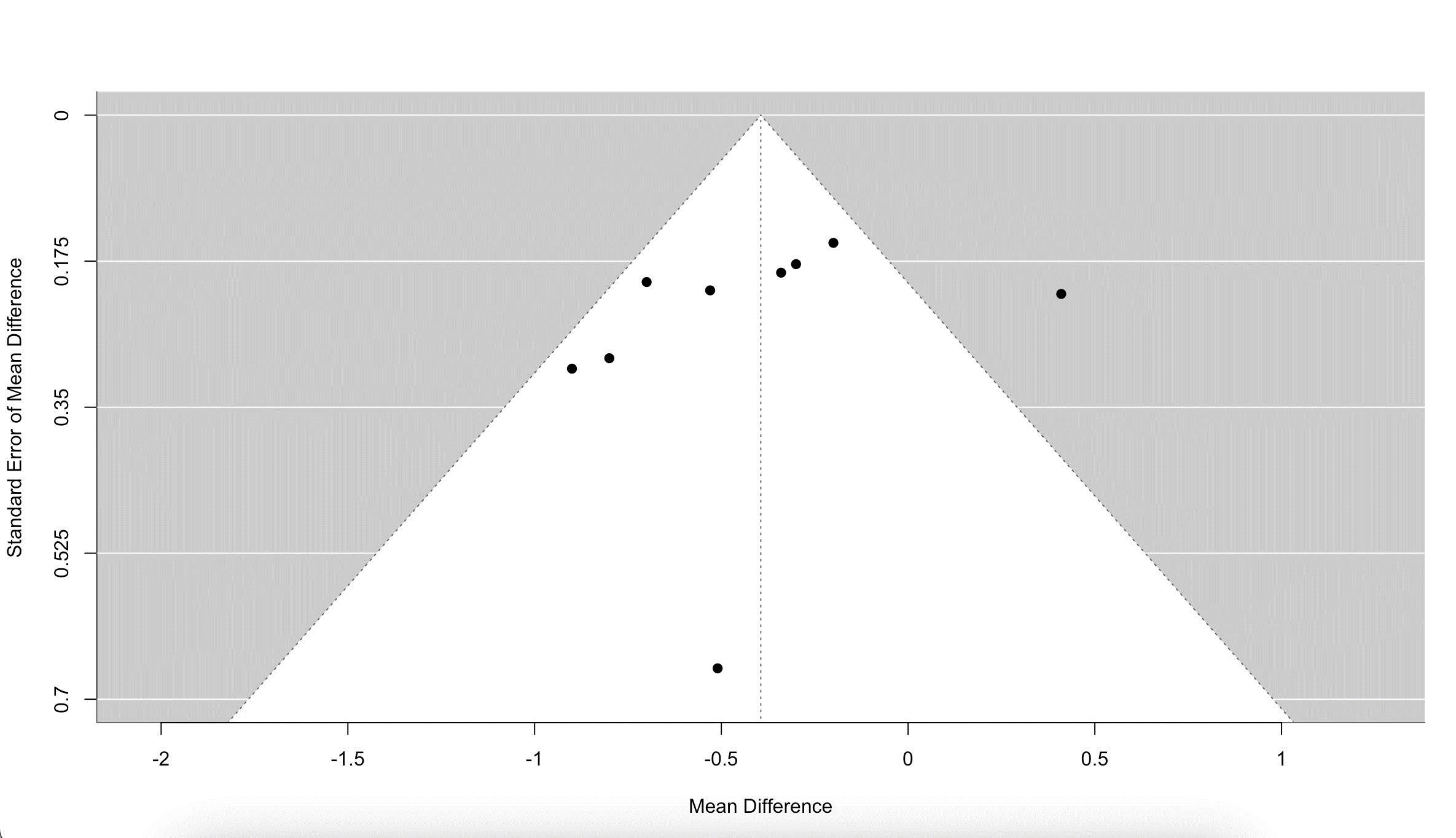


Supplemental Table 5. GRADE Strength of Evidence Assessment of Effect of Non-pharmacologic Interventions on Hemoglobin A1c in US Asian and Pacific Islander Populations

| Risk of Bias | Inconsistency | Indirectness | Imprecision | Publication Bias | Quality of Evidence |
| --- | --- | --- | --- | --- | --- |
| Moderate (lack of blinding) | No serious inconsistency | No serious indirectness | No serious imprecision | Undetected | ++++  High |
